# Explainable Artificial Intelligence in Assisted Reproductive Technology: Bridging Prediction and Clinical Judgment

**DOI:** 10.3390/biomedicines14051024

**Published:** 2026-04-30

**Authors:** Nektaria Kritsotaki, Dimitrios Diamantidis, Nikoleta Koutlaki, Nikolaos Machairiotis, Panagiotis Tsikouras

**Affiliations:** 1 Department of Obstetrics and Gynecology, Democritus University of Thrace, 68100 Alexandroupolis, Greeceptsikour@med.duth (P.T.); 2 Department of Urology, Democritus University of Thrace, 68100 Alexandroupolis, Greece; 3 Third Department of Obstetrics and Gynecology, University General Hospital “ATTIKON”, Medical School, National and Kapodistrian University of Athens, 12462 Athens, Greece; nikolaosmachairiotis@gmail.com

**Keywords:** explainable artificial intelligence, assisted reproductive technology, in vitro fertilization, embryo selection, machine learning, deep learning, SHAP, LIME, Grad-CAM, clinical decision support

## Abstract

**Background/Objectives**: Artificial intelligence (AI) models are increasingly applied across the assisted reproductive technology (ART) workflow, including male-factor assessment, ovarian stimulation, endometrial receptivity evaluation, embryo selection and prediction of pregnancy outcomes. However, many systems remain difficult to interpret, raising concerns regarding transparency, clinical integration and patient communication. Explainable artificial intelligence (XAI) aims to address these limitations by making model behavior more accessible to clinicians and embryologists. This review aimed to provide a narrative, concept-driven synthesis of how XAI has been implemented in ART, to critically examine methodological quality and clinical relevance and to outline priorities for responsible translation into practice. **Methods**: A structured narrative review was conducted using PubMed/MEDLINE as the primary database, supplemented by targeted reference-list screening of key primary studies and recent cross-disciplinary reviews relevant to AI in ART. Studies were curated and classified according to stage of the ART workflow, data modality, model family, explanation technique and validation strategy. Methodological features, performance reporting and implementation considerations were qualitatively appraised. **Results**: Most XAI applications in ART fall into two dominant categories: (i) feature-attribution methods such as SHAP and LIME applied to tabular clinical and laboratory data and (ii) saliency-based approaches, including Grad-CAM and related techniques, applied to embryo and ultrasound imaging. These methods can improve transparency and support counselling by clarifying which variables or image regions influence predictions. However, the majority of studies are retrospective and single centre, with limited external validation and heterogeneous outcome definitions, often prioritising clinical pregnancy over live birth. Calibration, decision-analytic evaluation and prospective assessment remain uncommon. XAI outputs are frequently interpreted as biologically causal despite being derived from observational data, highlighting the need for cautious clinical framing. **Conclusions**: XAI in ART has progressed from proof-of-concept demonstrations to early clinically oriented tools, but robust validation, standardised reporting and thoughtful workflow integration are still needed. Explanations can enhance auditability and communication, yet they do not compensate for methodological weakness. Future progress will depend on higher-quality multi-centre data, evaluation beyond discrimination metrics and governance frameworks that ensure transparency, fairness and sustained performance in real-world practice.

## 1. Introduction

Assisted reproductive technologies (ART), including in vitro fertilisation (IVF) and intracytoplasmic sperm injection (ICSI), are increasingly relied upon to manage infertility, which affects an estimated 10–15% of reproductive-aged couples worldwide. Despite technological advances, ART success rates remain modest and heterogeneous, constrained by biological variability as well as subjective clinical and embryological decision-making [1]. Traditional embryo assessment and selection protocols largely depend on visual grading and morphological criteria, which are time-consuming and prone to inter- and intra-observer variability [2,3].

Artificial intelligence (AI) and deep learning have emerged in ART as powerful tools capable of handling large, complex datasets and uncovering patterns beyond clinician perception. Studies have shown that AI can achieve promising performance in embryo morphology classification and clinical outcome prediction, in some settings approaching or exceeding expert-level performance [3]. Deep convolutional neural networks and fusion models that combine image and clinical data have shown promising discrimination for implantation and pregnancy outcomes [4,5]. However, many of these AI systems are “black boxes”, producing scores without transparent reasoning or human-intelligible explanations of how predictions are derived, a limitation that is particularly important in high-stakes clinical settings [6]. In embryo selection specifically, this shift from black-box prediction toward more transparent or “glass-box” approaches has become increasingly important for interpretability and clinical trust [7].

Explainable artificial intelligence (XAI) seeks to address this opacity by providing methods that elucidate model decision pathways and rationales. Techniques such as SHAP [8] and LIME [9] provide feature attribution for tabular clinical predictors, helping to contextualise which variables most influence a prediction (ISO explainable AI; SHAP/LIME definitions). In imaging domains, saliency-based methods such as Grad-CAM [10] enable visualisation of image regions that drive model outputs, offering clinicians insight into the spatial cues influencing predictions [11]. XAI frameworks are increasingly applied in other medical domains to ensure reliability, fairness and clinical trustworthiness, strengthening the interface between automated systems and human decision-makers.

The need for explainability in ART is not merely technical. Embryo selection and prediction of cycle outcomes involve ethically sensitive and high-stakes decisions affecting patient counselling, treatment burden, financial cost and emotional outcomes. Embedding explainability into ART models may foster clinician trust, facilitate shared decision-making with patients and identify potential biases or failure modes before deployment [12]. Although generic AI reviews in reproductive medicine and embryology describe broad applications of AI in IVF and laboratory practice [1,13,14,15,16], few specifically focus on how explainability is operationalised across the ART workflow.

This review systematically maps the current landscape of explainable AI in ART, from pre-treatment and male factor assessment through ovarian stimulation, endometrial evaluation, embryo scoring and cycle-level outcomes. We examine the main families of XAI methods employed, their practical implementations, validation strategies and limitations, and we discuss ethical and governance considerations that are crucial for translating explainability from research prototypes into responsible clinical tools.

Unlike broader AI-in-ART reviews, the present review is organised specifically around explainability across the ART workflow, integrating method typology, validation patterns and clinical translation challenges within a single XAI-focused framework.

## 2. Materials and Methods

A targeted literature search was conducted in PubMed/MEDLINE to identify studies on explainable artificial intelligence (XAI) in assisted reproduction published up to 31 December 2025. The search combined assisted reproduction terms “IVF”, “ICSI”, “assisted reproduction”, “ART”, “embryo”, “blastocyst”, “embryo selection”, “embryo grading”, “time-lapse”, “morphokinetics”, “ovarian stimulation”, “controlled ovarian stimulation”, “trigger day”, “endometrium”, “endometrial receptivity”, “endometrial thickness”, “sperm”, “semen”, “andrology”, “male factor”, “sperm DNA fragmentation”, “DFI” with artificial intelligence terms “artificial intelligence”, “machine learning”, “deep learning”, “neural network”, “convolutional neural network”, “CNN”, “random forest”, “gradient boosting”, “XGBoost”, “support vector machine” and “AutoML” and explainability terms “explainable AI”, “interpretability”, “feature attribution”, “feature importance”, “SHAP”, “LIME”, “permutation importance”, “saliency map”, “class activation map”, “CAM”, “Grad-CAM”, “ScoreCAM”, “layer-wise relevance propagation”, “attention”, “counterfactual”, “latent space” and “disentangled”. Searches were supplemented by reference list screening of key articles to capture influential ART decision-support models that may be inconsistently indexed. Records were screened first at title and abstract level for relevance to XAI in ART, followed by full-text review of potentially eligible articles to confirm clinical scope, methodological relevance and the presence of an explainability component. To reduce the risk of missing technically indexed literature, we also considered recent cross-disciplinary reviews in AI and reproductive medicine that had searched broader sources such as Scopus, IEEE Xplore, ACM Digital Library, Embase and Web of Science.

Studies were considered relevant if they applied artificial intelligence or machine learning to a clinically meaningful task in assisted reproduction and reported an explainability component intended to support clinical interpretation. Eligible application areas included embryo assessment and selection, prediction of ovarian stimulation response and trigger timing support, evaluation of the endometrium and receptivity and cycle-level prediction of pregnancy or live birth. Andrology studies were included when the model addressed male factor questions in the ART pathway and provided an interpretable output suitable for counselling or clinical decision support. To reflect the focus of this review, included studies were required to report at least one of the following explainability elements: feature attribution for tabular models, including SHAP- or LIME-based outputs; visual explanation for image-based models, including saliency or CAM variants; or interpretability by design, such as rule-based models or clinician-readable scoring systems. Highly influential ART prediction models without an explicit explainability layer were considered only when necessary to contextualise how XAI is being positioned for clinical adoption. We excluded studies that did not address a clinically meaningful ART task, did not contain an interpretable or explainability-related component, were not sufficiently described to allow qualitative appraisal or fell outside the ART workflow considered in this review. Purely technical papers without clear reproductive relevance and studies focused only on conventional AI performance without interpretability were not retained unless needed for conceptual context.

Given the narrative scope and the substantial heterogeneity in study design, endpoints, data modalities and explainability approaches, we did not apply a formal risk-of-bias tool. Instead, evidence was appraised qualitatively with emphasis on clinical endpoints, validation strength, reporting transparency, overfitting risk and whether performance reporting extended beyond discrimination to include calibration or clinically interpretable operating thresholds.

## 3. Conceptual and Regulatory Foundations of XAI in ART

In clinical machine learning, explainable AI (XAI) describes approaches that render model behaviour sufficiently transparent to be understood, scrutinised and, when needed, challenged by clinicians and patients, rather than accepted as opaque “black-box” scores. In ART, this requirement is amplified because AI outputs can directly shape embryo selection, treatment burden, counselling and the perceived probability of pregnancy and live birth for individual couples [12,16,17,18]. In practice, most XAI implementations in reproductive medicine fall into a small set of recurring method families. For tabular cycle-level prediction and clinical decision support, feature attribution techniques such as SHAP and LIME dominate, providing global patterns of predictor influence and patient-specific explanations that can support counselling and contestability [19,20,21,22,23,24]. For image-based systems, particularly embryo imaging and endometrial assessment, saliency methods and class activation mapping approaches such as Grad-CAM/ScoreCAM and related relevance techniques are widely used as a minimum layer of visual transparency by highlighting regions that most strongly drive a prediction [4,12,25]. Beyond these, newer work explores counterfactual and generative explanations, including models that structure embryo appearance into interpretable latent factors that can be adjusted along specific morphological axes, as well as inherently interpretable tools such as rule-like clinical frameworks and constrained regression-style models that can be read directly and, when helpful, supplemented by attribution plots [19,26,27,28].

Alongside methodology, the governance landscape increasingly frames explainability as a practical requirement for deployment in ART. Broad health-AI guidance emphasises transparency, accountability, privacy protection, human oversight and equity, while in Europe, the combined regulatory environment is expected to treat embryo selection and reproductive decision-support systems as high-risk applications, with explicit expectations for risk management, robustness and provision of understandable information on how outputs are generated. Regulatory experience with AI-enabled devices also differentiates static (“locked”) models from adaptive systems, the latter raising additional demands for monitoring, post-deployment surveillance and real-world performance assessment. Within ART specifically, consensus guidance calls for high-quality data governance, rigorous validation, ideally beyond single-centre internal testing, independent assessment of commercial tools and explanations that are interpretable to clinicians and meaningful to patients [17].

Ethical considerations in ART are distinctive because AI operates on decisions about embryos, family building and the emotional burden of treatment. Concerns include misinterpretation of embryo-ranking outputs as categorical judgements of embryo “worth”, psychological harm from probabilistic predictions in a vulnerable population and inadvertent pressure toward additional cycles driven by model-derived counselling [17,29]. Afnan et al. emphasise that AI-based embryo ranking must be framed as an aid to informed choice rather than a tool that implicitly redefines what counts as a “better” embryo and argue for explicit safeguards against eugenic drift [30]. Bias and inequity are central risks when training data under-represent certain ethnic groups, ages, diagnoses or clinic settings, allowing apparently strong overall metrics to mask systematic underperformance in subgroups. Questions of data ownership and commercial reuse are also particularly acute in ART, where datasets frequently originate in private settings and are used to develop proprietary systems that remain difficult to audit. In this context, explainability is not a cosmetic add-on but a mechanism to support clinician oversight, detect bias and failure modes and communicate reasoning and uncertainty to patients in a way that preserves clinical responsibility. For high-stakes ART decisions, especially embryo ranking and treatment strategy, practical “good enough” explainability typically requires more than one layer of transparency, combining stable global patterns with case-level explanations and, for image-based tools, visual or structured representations that can be interpreted alongside embryologist or sonographer judgement [12,17,29].

## 4. Clinical Applications of Explainable AI Across the ART Pathway

### 4.1. Interpretable Modelling in Male-Factor Infertility Relevant to ART

Explainable and clinically interpretable modelling has been explored for pre-treatment risk stratification in male-factor infertility, spanning lifestyle-based proof-of-concept datasets and more clinically grounded predictors linked to embryo development. In the UCI Male Fertility dataset (100 men aged 18–36 years; 88 normal and 12 altered semen quality by WHO criteria), lifestyle and environmental variables were used to screen semen quality using explainable machine learning. One study compared seven standard classifiers, support vector machine, random forest, decision tree, logistic regression, naïve Bayes, AdaBoost and multilayer perceptron, with and without SMOTE oversampling of the minority class. Predictors included season of analysis, age, childhood disease, accident or trauma, surgical history, high fever in the previous year, alcohol intake, smoking status and daily sitting hours, with semen quality as a binary label. Under SMOTE, tree-based and ensemble models reported very high discrimination, with cross-validated accuracies above 90% and AUC values close to 1.0, and model behaviour was examined using SHAP, LIME and ELI5. Explanations consistently identified age as the dominant contributor, followed by season, recent febrile illness, alcohol consumption, smoking and sedentary time, whereas prior urogenital surgery had minimal impact on model output [24].

Using the same dataset, a second framework proposed a hybrid neural architecture coupled with ant colony optimisation and a proximity-based mechanism intended to create similarity-aware train–test partitions. After min–max rescaling, 71 samples (61 normal, 10 altered) were assigned to training and 29 to testing; on this split, the model reported 99% accuracy, 100% sensitivity and 98.87% specificity, with a Matthews correlation coefficient of 0.952. Explanations were presented as global feature-importance outputs again emphasising lifestyle and environmental exposures such as sedentary behaviour and recent fever, together with a similarity-based contextualisation of individual predictions by referencing clusters of comparable patients [31].

In a more clinically oriented retrospective study of 373 couples with male-factor infertility, a prediction model coupled with causal analysis was developed for the proportion of high-quality embryos using a predefined target of at least 45%. Logistic regression and causal modelling identified sperm DNA fragmentation index, male anthropometrics such as height and weight and mycoplasma infection as negative factors associated with achieving the high-quality embryo target. An intervention-style simulation suggested that treating infection, reducing DNA fragmentation and weight loss in overweight men were associated with improved high-quality embryo rates, providing a transparent “what-if” layer aligned with pre-treatment counselling and risk modification [32]. On the imaging side, convolutional neural networks have also been used for automated sperm morphology assessment, with Grad-CAM visualisations indicating that the models focus on acrosomal and head-shape regions rather than background artefacts, illustrating how XAI can be used to audit deep models in andrology workflows [33].

### 4.2. Ovarian Stimulation, Follicle Selection and Trigger Timing

#### 4.2.1. Interpretable Decision Support for Trigger Timing and Oocyte Yield

An intrinsically interpretable approach for timing the ovulation trigger in IVF has been proposed, using transparent clinician readable rules rather than post hoc explanation of a complex model. The framework relies on routinely available stimulation day inputs, particularly the distribution of follicles across predefined ultrasound size categories and serum estradiol concentrations, complemented by basic patient and cycle characteristics. Recommendations arise from a compact rule-based structure in which a small set of explicit if–then conditions combine follicle size patterns with estradiol thresholds. Each rule corresponds to a clinically recognisable scenario, such as reaching a specified number of follicles above a diameter cut-off within a defined estradiol range, and yields a direct recommendation on whether the trigger is appropriate on that day or whether stimulation should continue. In internal validation, performance for capturing the relationship between follicular response and downstream oocyte yield was comparable to more complex machine learning baselines, while the decision logic remained fully inspectable [28]. 

An interpretable machine learning model has also been developed to predict the number of mature oocytes retrieved after controlled ovarian stimulation at scale, using routine cycle variables with SHAP for explanation. In 24,976 IVF or ICSI cycles, feature selection converged on a small predictor set centred on antral follicle count, baseline gonadotropin and estradiol values, estradiol and large follicle counts on the trigger day and female age. The best performing model showed good discrimination and calibration in an independent temporal dataset, and SHAP prioritised trigger day estradiol and the number of large follicles as the main drivers of prediction. A web-based calculator was provided to support individualised counselling [34].

#### 4.2.2. XAI for Follicle-Level Optimisation

Building on these cycle-level tools, an explainable gradient boosting model has been used to optimise stimulation at the follicle cohort level by treating the trigger day follicle size distribution as the main predictor of oocyte yield and IVF outcomes in a large multicentre dataset. In 19,082 treatment-naive women across 11 clinics, follicle diameters from 6 to 26 mm were recorded as counts per millimetre and linked to retrieved oocytes, metaphase II oocytes, two pronuclear zygotes, good quality blastocysts and live birth after fresh transfer. Prediction of metaphase II oocyte number from the follicle size histogram achieved a mean absolute error of about 3.6 oocytes with R^2^ of 0.35, and adding basic clinical variables offered only marginal gains. Interpretability was provided using permutation importance and TreeSHAP, with the most informative signal concentrated in an intermediate follicle band, approximately 13 to 18 mm for metaphase II oocytes and 14 to 20 mm for good quality blastocysts. These explanations were translated into range-based trigger criteria defined by the proportion of follicles within the optimal band. A higher proportion of 13 to 18 mm follicles was also associated with higher live birth, whereas larger mean follicle size and higher progesterone aligned with lower live birth without meaningful improvement in mature oocyte yield [35].

#### 4.2.3. Cycle-Level Pregnancy Prediction with Explainability

Several studies have applied explainable machine learning to cycle-level prediction of IVF or ICSI outcomes using routine clinical and stimulation variables as inputs, with SHAP or LIME used to clarify which factors drive model outputs. Web-based implementations have been described with separate models for the pre-treatment stage and the treatment phase, combining risk estimates with SHAP visualisations. The pre-treatment model relies on age, ovarian reserve markers and baseline characteristics, while the treatment-phase model additionally incorporates stimulation parameters, hormone profiles and embryo counts. Across both phases, SHAP consistently prioritises familiar predictors such as female age, AMH, progesterone on the trigger day, number of retrieved oocytes and number of transferable embryos and quantifies how these variables combine within an individual cycle to shift predicted probability of pregnancy [19].

Explainability has also been used around the embryo transfer decision, including models that integrate demographic factors, stimulation characteristics, embryo morphology and self-reported nutritional supplement use. LIME has been applied to generate case-specific explanations, indicating for each patient whether variables such as age, body mass index, endometrial thickness, embryo quality or supplement exposure are driving the predicted probability. In this setting, explanation outputs should be interpreted as descriptions of model behaviour within observational data rather than evidence of causal effects [23]. Subgroup-specific modelling has been reported in women with polycystic ovary syndrome, where an XGBoost model with SHAP explanations was used to predict live birth after IVF or ICSI. Features included anthropometry, metabolic and androgen profiles, ovarian reserve markers and stimulation outcomes. SHAP plots prioritised body mass index, hyperandrogenism, AMH, age and oocyte yield and suggested that adverse metabolic status attenuates the apparent benefit of favourable ovarian reserve or oocyte number on live birth [21].

Across cycle-level prediction studies in ART, explainability improves transparency of individualised predictions but does not, by itself, address confounding, selection bias, limited external validation or incomplete reporting of calibration and clinical utility. These limitations remain important when translating SHAP- and LIME-based explanations into counselling or decision support [16,18].

### 4.3. Endometrium and Uterine Environment: Ultrasound and Disease-Specific XAI

A prospective single-centre study linked multimodal transvaginal ultrasound findings obtained the day before embryo transfer with ongoing pregnancy after IVF. Eighty-six women underwent a standardised protocol combining two-dimensional endometrial assessment, Doppler-based blood flow grading, three-dimensional power Doppler angiography and contrast-enhanced ultrasound with extraction of volumetric vascular indices and quantitative time intensity parameters from the endometrium and subendometrium. The endpoint was ongoing intrauterine pregnancy beyond 14 weeks of gestation. After LASSO-based feature selection, several classifiers were evaluated, and gradient boosting performed best, with internal accuracy around 93% and an AUC close to 0.98. SHAP was then used to rank predictors and illustrate directionality, with higher numbers of MII oocytes and more favourable blood flow grades increasing predicted probability, while larger uterine cavity volume and higher endometrial or subendometrial peak intensities on contrast-enhanced ultrasound decreased it. Case-level SHAP force plots showed how these variables combined to push predictions above or below baseline risk. Given the small sample size, the reported performance should be interpreted cautiously and warrants external validation [36].

An explainable cycle-level model has been developed to predict clinical pregnancy in women with surgically confirmed endometriosis undergoing fresh IVF or ICSI with embryo transfer. In 1752 patients, six machine learning models were trained using 24 routine variables covering female and male characteristics, stimulation details, hormone measurements, fertilisation data and embryo transfer parameters. XGBoost performed best on an independent test set, with an AUC of approximately 0.62 and moderate accuracy, consistent with the known difficulty of prognostication in this population. SHAP analyses prioritised progesterone on the trigger day, AMH, number of embryos transferred, number of normal fertilisations, female age and BMI as the main drivers of prediction, while many other stimulation and semen variables contributed minimally. Case-level SHAP force plots highlighted, for each woman, whether elevated progesterone, low AMH, limited normal fertilisation or single embryo transfer were the dominant factors lowering the predicted probability of pregnancy [20].

### 4.4. Embryo Scoring, Ploidy and Embryo Selection

#### 4.4.1. CNN Embryo Models with Saliency and Relevance Maps

Several image-based embryo selection systems now combine convolutional neural networks with visual explanation tools, aiming to move beyond opaque score-only outputs. An early convolutional framework used multifocal embryo images to jointly perform segmentation, developmental stage classification and blastocyst grading and applied class activation maps to visualise which regions of the embryo most influenced the predicted score [37]. A ResNet 18 model trained on 19,342 static day 5 blastocyst images after single embryo transfer was developed to predict clinical pregnancy, with a secondary analysis of live birth. The image-only model achieved an AUC of 0.68 for clinical pregnancy in the test subset, compared with 0.62 for a control network trained on Gardner grades alone, while an ensemble combining the image score with clinical variables such as age, AMH, transfer day hormones, endometrial thickness and BMI reached an AUC of 0.71. Grad CAM heatmaps highlighted compact inner cell mass and cohesive trophectoderm in embryos that implanted and more heterogeneous fragmented regions in non-implanting blastocysts, in patterns presenting as concordant with embryologist interpretation. In the ensemble, SHAP ranked the image score as the dominant predictor, followed by female age, pregnancy history, AMH, and transfer day estradiol and progesterone [25].

A second multimodal deep learning system fused a ResNet 34 image branch with a multilayer perceptron for clinical data, training separate models for clinical pregnancy and live birth. For clinical pregnancy, the clinical-only model achieved an AUC of 0.91, the image-only model 0.73 and the fusion model 0.91, while for live birth, the corresponding AUCs were 0.87, 0.80 and 0.88. ScoreCAM maps suggested that well-performing image models focus on the trophectoderm and embryo contour but also revealed cases where attention drifted to background regions, indicating unreliable decision patterns. In related work, a compartment-aware CNN treated inner cell mass and trophectoderm as partially separate inputs and used saliency maps to show whether decisions were driven predominantly by ICM- or TE-dominant regions, aligning explanations more closely with established morphological criteria [38]. Layer-wise relevance propagation on the clinical branch highlighted female age, male age, sperm motility and female BMI as leading contributors. A Bayesian reliability classifier based on saliency distributions achieved modest discrimination between correct and incorrect predictions [4]. Other high-performing convolutional and morphokinetic models for embryo classification and miscarriage risk prediction, including recent time-lapse deep learning systems trained on matched high-quality embryos, provide little or no explicit explanation beyond a score, underscoring the gap that saliency and relevance methods aim to address [39,40,41].

#### 4.4.2. Time Lapse Embryo Ranking with Hybrid XAI (Video + Clinical Context)

A time lapse-based embryo prediction framework has been reported that combines a deep video-derived embryo score with routine clinical variables into a hybrid model designed for contextualised embryo ranking. The system uses a morphokinetic representation learned from embryo videos and integrates it with cycle-level features in a gradient boosting layer, allowing the final prediction to reflect embryo quality in the specific clinical setting of the couple rather than as an isolated image score. Performance was evaluated across multiple clinics with clinic hold-out experiments, and the hybrid approach showed moderate discrimination with variability by site, consistent with differences in missingness and local practice. In a direct comparison against senior embryologists on a held-out test set, the model achieved higher specificity and modest gains in balanced accuracy and related summary measures, while embryologists remained slightly more sensitive at the chosen operating threshold.

Explainability was provided at the hybrid layer using SHAP, which consistently ranked the video score as the dominant driver while also assigning non-trivial contributions to clinical factors spanning ovarian response, endometrial features and semen parameters. This structure enables embryo ranking that is explicitly adjusted by patient level context, and the SHAP outputs provide a clinician-readable account of how the same embryo score can translate into different predicted outcomes depending on age, stimulation response and uterine environment [26].

#### 4.4.3. Interpretable Embryo Scoring and Selection Tools

A retrospective interpretable deep learning model has been developed to quantify blastocyst morphology beyond categorical Gardner grading by producing continuous scores for inner cell mass and trophectoderm quality and combining them into a single score linked to live birth. The model was trained on 2760 blastocysts with majority-voted grades and then applied to 15,228 blastocysts with known live birth outcomes, showing a clear monotonic association between higher inner cell mass and trophectoderm scores and higher live birth rates. Importantly, the scoring approach remained interpretable because it retained the Gardner framework as the clinical reference and expressed output as continuous, clinician-readable measures rather than a single opaque probability. External application in a second institution showed consistent correlation of the combined score with live birth, supporting generalisability across imaging conditions and embryologist grading styles [42].

An explainable embryo selection framework using static blastocyst images has been proposed on a small public dataset, combining a deep classifier with local, image-level explanations based on LIME superpixel perturbations. For each embryo, the explanation layer highlights the specific image regions that support a good versus poor classification, allowing visual inspection of whether the network focuses on clinically plausible structures. The study also reported quantitative interpretability measures, including high local fidelity of the surrogate explanation and substantial overlap between highlighted regions and expert annotated embryo areas while explicitly discussing the clinical risk of false positive classification of poor embryos as good. Although the endpoint is morphological quality rather than implantation or live birth, it provides a clear example of local, case-specific explainability on embryo images beyond saliency maps [43].

Prospective evaluation has also been reported for an interpretable AI-assisted embryo selection tool in a single-centre cohort of 250 fresh single blastocyst transfers, where patients chose between an AI-assisted pathway in which embryologists followed the model ranking and a manual pathway based on Gardner grading alone. Implantation rate was higher in the AI-assisted group at 80.87% versus 68.15% with p equal to 0.022, with no observed differences in miscarriage, live birth or neonatal outcomes [44].

#### 4.4.4. Ploidy and PGT-A-Related XAI

Explainable modelling has been applied to blastocyst ploidy prediction by combining routine clinical variables with time lapse-derived morphokinetic features. In this setting, gradient boosting classifiers were interpreted using SHAP to define global feature importance and LIME to provide case-level explanations. Maternal age, embryo development timing and blastocyst quality emerged as dominant predictors, and SHAP dependence plots showed clinically intuitive patterns, including lower predicted aneuploidy risk in embryos from younger women with timely blastulation and favourable morphology. LIME outputs were presented as embryo-specific explanation reports, indicating which clinical and morphokinetic features most strongly supported a euploid or aneuploid prediction [45].

Explainability has also been used downstream after PGT A, focusing on biochemical pregnancy loss following single euploid embryo transfer. A random forest model integrating maternal characteristics, stimulation parameters, endometrial preparation data and embryo quality metrics was interpreted with SHAP, which prioritised a small set of drivers distinguishing biochemical loss from progression to clinical pregnancy. These predictors were reported to align with recognised risk factors and to point toward potentially modifiable elements of endometrial preparation and cycle management [46].

#### 4.4.5. Generative and Latent-Space XAI for Embryo Morphology

Beyond saliency maps on raw images, a generative approach has been introduced to separate embryo morphology into a small number of clinically meaningful visual factors and to support counterfactual style explanations. A disentangled latent representation is learned from large collections of blastocyst images, so that individual latent dimensions correspond to interpretable embryo traits rather than mixed, entangled features. In this framework, predictions are made from the latent coordinates, and explanation is performed at the level of these coordinates rather than at the pixel level. Feature attribution methods can then quantify which latent traits are most influential for a given embryo, and controlled editing along a single latent direction can generate synthetic counterfactual images illustrating how the model score would change if one morphological factor improved or worsened while others remain stable [47].

A complementary direction aims for interpretability by constructing embryo representations from measurable, clinician-familiar features rather than from purely implicit image embeddings. In this setting, image-based pipelines extract structured embryo descriptors that can be inspected directly and then used for prediction, allowing explanations to be expressed in terms that align with embryologist assessment rather than heatmaps or abstract latent axes [27].

### 4.5. Cycle-Level and Disease-Specific Outcome Prediction

Beyond stimulation-focused models, several explainable frameworks address cycle-level prediction of pregnancy or live birth across general IVF and ICSI populations as well as selected disease-specific cohorts. One approach described an LLM-assisted AutoML workflow in which a large language model proposes and configures candidate models on IVF clinical datasets, with selection converging on an XGBoost classifier that is subsequently interpreted using SHAP. The system outputs an individualised probability of pregnancy together with a ranked set of drivers such as female age, AMH, embryo number and endometrial thickness and generates a brief narrative explanation derived from the SHAP pattern [22].

Explainability has also been embedded in web-based counselling tools through pre-treatment and in-cycle prediction models for IVF and ICSI that display SHAP plots alongside risk estimates. Adaptations to specific phenotypes include an endometriosis-focused XGBoost plus SHAP model for clinical pregnancy in 1752 fresh transfer cycles, in which progesterone on the trigger day, AMH, fertilisation measures and embryo load emerged as key drivers. In PCOS, an XGBoost model with SHAP explanations for live birth prioritised BMI and hyperandrogenism and suggested that metabolic status modifies the apparent benefit of favourable ovarian response and higher oocyte yield [19,20,21].

Other frameworks emphasise particular biological compartments. Advanced transvaginal ultrasound and contrast-enhanced perfusion parameters of the endometrium and subendometrium have been integrated into a gradient boosting model interpreted with SHAP for ongoing pregnancy, making explicit how blood flow grading and quantitative perfusion indices combine with oocyte yield in the prediction. At the embryo transfer stage, models have also incorporated self-reported nutritional supplement use as a potentially modifiable exposure, using LIME to show at the individual level whether supplement variables, BMI, endometrial thickness or embryo morphology were most influential for the predicted probability [23,36].

**Figure 1:**
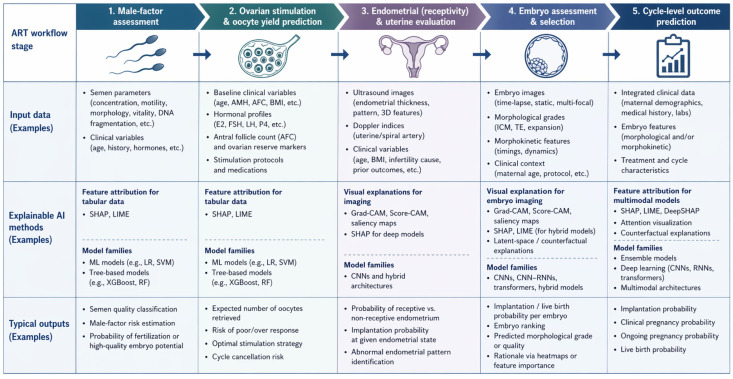
Explainable artificial intelligence across the assisted reproductive technology pathway. Conceptual overview of the main application domains of XAl in ART. For each stage, representative input data, commonly used explainability approaches and typical clinical outputs are shown. Abbreviations: AFC, antral follicle count; AMH, anti-Müllerian hormone; BMI, body mass index; CNN, convolutional neural network; E2, estradiol; FSH, follicle-stimulating hormone; ICM, inner cell mass; LR, logistic regression; LH, luteinizing hormone; LIME, local interpretable model-agnostic explanations; ML, machine learning; P4, progesterone; RF, random forest; RNN, recurrent neural network; SHAP, Shapley additive explanations; SVM, support vector machine; TE, trophectoderm.

Across these cycle-level approaches, SHAP and LIME repeatedly highlight canonical predictors including female age, AMH, progesterone on the trigger day, embryo number and quality and endometrial thickness while also making less routinely used signals such as metabolic markers, supplement exposure and Doppler- or contrast-enhanced indices more explicit for patient-specific counselling [19,20,21,23,36]. Because these models are observational and often lack robust external validation, explanation outputs should be interpreted as structured associations within the analysed data rather than as causal prescriptions [16,18]. To synthesise the evidence reviewed in this section, Figure 1 summarises the main application domains of explainable artificial intelligence across the ART workflow, together with representative inputs, explainability approaches and typical clinical outputs.

Additionally, to provide an overview of the evidence base summarised in this section, the included studies are mapped below across the main ART decision points, indicating the underlying data modality, endpoint, explainability approach and validation setting (Table 1 and Table 2).

## 5. Typology of XAI Methods Used in ART

### 5.1. Feature-Attribution Methods for Tabular Data

Most explainable ART models built on clinical, laboratory or stimulation variables use post hoc feature attribution to show which predictors drive the output. SHAP is used most often across cycle-level outcome models and disease specific cohorts and typically ranks familiar determinants such as female age, AMH, progesterone on the trigger day, oocyte and embryo counts, BMI and metabolic markers, while also bringing less routinely discussed signals into view, including sperm-related variables and, in selected studies, Doppler- or contrast-enhanced ultrasound indices. Global SHAP summaries describe the overall hierarchy of predictors, while case-level plots show which variables move an individual estimate up or down [19,20,21,24,36,46]. 

LIME is reported less frequently and is mainly used when short, case-specific explanations are emphasised, including embryo transfer outcome and ploidy-related prediction. ELI5 appears sporadically as a supplementary importance tool [23,24,45]. These methods support clinical interpretability but remain sensitive to model choice and collinearity, and when applied to observational datasets, they can encourage over-interpretation of non-causal associations unless validation and clinical scrutiny are rigorous [18]. A small number of conceptual proposals also explore LLM-assisted pipelines in IVF datasets, where language models suggest model architectures that are subsequently interpreted with SHAP, positioning XAI both as a counselling tool and as an aid to clinician-led model development [48].

### 5.2. Visual Explanation for Embryo Imaging

In embryo image models, explainability is usually provided through saliency or CAM-style visualisations layered onto CNN predictions. Grad CAM and ScoreCAM are used to highlight image regions associated with implantation or viability scoring, and multimodal systems may pair image heatmaps with relevance methods on the clinical branch to show which non-image variables matter. These displays are intuitive for embryologists but can be unstable, can focus on background artefacts and do not reliably map to discrete biological concepts, so they should be interpreted as visual plausibility checks rather than mechanistic evidence [4,12,25].

### 5.3. Interpretability by Design

Alongside post hoc explanation, some approaches aim for transparency at the level of model design. A trigger-timing model based on simple combinations of follicle size distributions and oestradiol levels, expressed as explicit rules rather than opaque network weights, provides cycle-level recommendations that can be read and critiqued directly by clinicians [28]. At the embryo level, day-3 models that restrict inputs to a small set of human-readable morphological and kinetic features and expose their contributions explicitly illustrate how embryo assessment can be aligned with simpler, more interpretable representations without fully abandoning predictive performance [49].

Hybrid strategies include logistic or regression models with restricted splines, where the functional form remains relatively transparent, but SHAP overlays are used to visualise non-linear effects and to provide case-level explanations [19]. These designs occupy a middle ground between fully black-box architectures and hand-crafted scoring systems and may be more acceptable in settings where auditability and straightforward communication are prioritised.

### 5.4. Latent Trait and Counterfactual Explanations for Embryo Morphology

**Figure 2:**
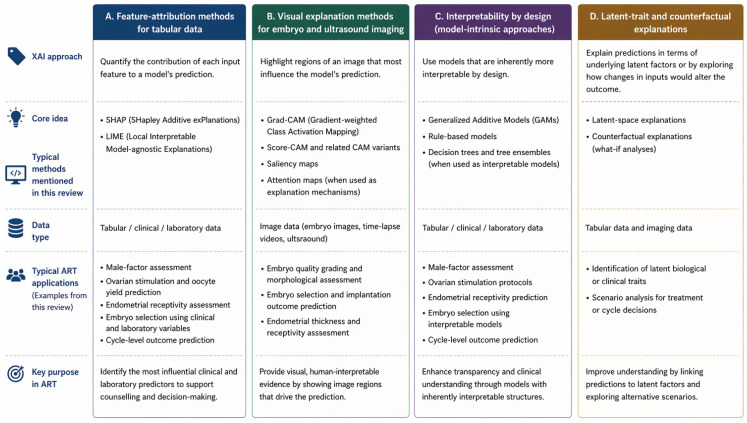
Typology of explainable artificial intelligence methods used in assisted reproductive technology. Schematic summary of the main families of explainable artificial intelligence (XAI) discussed in this review, including feature-attribution, visual explanation, interpretability-by-design and latent-trait or counterfactual approaches. Abbreviations: ART, assisted reproductive technology; CAM, class activation mapping; Grad-CAM, gradient-weighted class activation mapping; LIME, local interpretable model-agnostic explanations; SHAP, Shapley additive explanations.

More structured explanations have been proposed for embryo morphology by moving from pixel heatmaps to trait-level representations. Disentangled latent spaces allow embryo appearance to be expressed along a small number of morphology axes, with prediction performed from these coordinates and explanation framed in terms of which traits drive the score. The same representation can support controlled editing along one axis at a time to illustrate counterfactual changes in predicted outcome. This approach differs from CAM methods by explaining embryo level attributes rather than attention patterns, but its clinical role depends on prospective validation and integration into decision workflows [27,47]. Other frameworks attempt to couple performance optimisation and explanation in a single pipeline, aiming to stabilise feature importance and reduce the mismatch between training objectives and interpretability outputs [50]. To summarise the methodological families discussed in this section, Figure 2 synthesises the main explainable artificial intelligence approaches used in ART, including feature-attribution methods, visual explanation methods, interpretability-by-design approaches, and latent-trait or counterfactual explanation strategies.

Taken together, these method families offer different kinds of transparency rather than a single hierarchy of explainability. Feature-attribution methods such as SHAP and LIME are most useful in tabular clinical models, where they support case-level counselling and global inspection of predictor influence, but they remain vulnerable to collinearity and over-interpretation of non-causal associations. In contrast, CAM- and saliency-based approaches are intuitive for embryo imaging because they provide visual overlays, yet they are less stable and should be interpreted as plausibility checks rather than direct biological explanations. Interpretability-by-design approaches offer greater auditability and easier clinical communication, whereas latent-trait and counterfactual frameworks are promising for richer embryo-level explanation but remain less mature for routine deployment.

## 6. Methodological Quality, Validation and Generalisability

### 6.1. Study Design and Data Sources

Across the ART AI literature, most models are retrospective, single centre and evaluated with internal splits, with heterogeneous endpoints and variable reporting. The XAI papers largely follow the same pattern. Tabular models for cycle prediction and disease-specific prognosis are usually derived from one or a few clinics and range from several hundred to a few thousand cycles. Image-based embryo models typically use retrospective datasets of several thousand blastocyst images from a single laboratory or network, again with internal validation only [4,12,16,18,19,20,25].

More rigorous designs are reported but remain uncommon. Examples include phenotype-aware modelling with explicit separation of pre-treatment and in-cycle prediction, partial external testing for ploidy prediction, prospective collection for multimodal endometrial imaging and one of the few prospective, trial-like evaluations of an interpretable embryo ranking strategy against standard morphology. These studies illustrate feasibility, but they are exceptions rather than the dominant design pattern [19,44,45].

Across the studies reviewed, validation strength remained uneven. Most models were developed in retrospective single-centre datasets and evaluated using internal train-test splits or cross-validation, whereas temporal validation, partial external validation, and prospective clinical evaluation were distinctly less common [4,16,18,19,25,34,44,45]. This distinction is clinically important, because apparently strong discrimination in internally validated studies may not translate into stable performance across different clinics, imaging systems, laboratory practices or patient populations [16,18]. Reporting of calibration and clinical utility was also inconsistent, which limits interpretation of whether model outputs are suitable for individual risk estimation or for actual decision support in routine ART practice [16,18,21,34].

### 6.2. Outcomes and Performance Reporting

Clinical pregnancy is the most commonly reported endpoint, with implantation, biochemical pregnancy or early clinical pregnancy frequently used, while live birth is less often the primary outcome and is sometimes reported only secondarily. Heterogeneous outcome definitions limit comparability and can favour endpoints that are easier to predict but less meaningful for patients. Performance reporting is usually centred on discrimination. Only a minority of studies extended evaluation beyond discrimination to include calibration or decision-analytic assessment, and even fewer combined these measures with stronger forms of validation such as temporal, external or prospective evaluation [16,18,21,34]. AUC and accuracy dominate; sensitivity and specificity are inconsistently reported, and predictive values are rarely emphasised despite their relevance for counselling. Calibration and clinical utility assessments remain uncommon. As a result, even models with acceptable discrimination may provide poorly characterised absolute risk estimates and uncertain clinical benefit [16,18].

### 6.3. Overfitting and Bias

Several studies illustrate overfitting risks, particularly where small datasets are paired with complex learners, extensive feature sets, oversampling or optimisation schemes, with very high reported performance that is unlikely to transport to new settings. Similar concerns apply when event rates are low relative to model complexity and when external validation is absent. Publication bias is also likely, as negative or neutral models are rarely presented, and incremental gains are often framed as superiority without clear uncertainty quantification [16,18,24,31].

### 6.4. XAI Specific Pitfalls

Explainability outputs are commonly interpreted more strongly than warranted. SHAP and LIME describe model behaviour within observational data and should not be read as causal effects; high attribution to modifiable factors such as BMI or supplement exposure does not imply that intervention will change outcomes as predicted. In imaging, saliency and CAM methods can highlight artefacts or background, and visually compelling plots may encourage over-trust, particularly when paired with automated narratives, even in weakly validated models. Their apparent precision may exceed their true reproducibility, especially when predictor collinearity, dataset shift or weak validation are present [4,12,19,23].

Overall, XAI in ART should be judged under the same standards as any clinical prediction modelling: appropriate design, adequate sample size, transparent reporting and validation that supports generalisability. Explanations can support scrutiny and communication, but they do not compensate for methodological weakness [16,18].

## 7. From Algorithm to Clinic: Implementation, Ethics and Governance

### 7.1. Workflow Integration and Human Factors

Successful use of AI in ART depends less on model architecture and more on whether the tool fits the daily reality of the clinic and laboratory. Interfaces need to be simple, reliable and embedded in existing workflows, with clear instructions on when an output should be consulted and how it should be weighed against clinical judgement. Co design with embryologists and clinicians, standardised training and documented escalation pathways for discordant cases help prevent both over-reliance and silent nonuse [12,17]. Examples of pragmatic integration include counselling tools that present a risk estimate together with a brief explanation, while keeping the clinician in control of interpretation and communication. In the laboratory, explainable embryo ranking needs to align with routine grading and documentation, with explicit local rules for how AI rankings and embryologist assessment are combined and how disagreements are handled [4,12,19,25].

### 7.2. Patient Communication and Shared Decision-Making

Explainability can support transparent counselling if it is framed as an aid to understanding, not as a verdict. Case-level explanations can be translated into plain language statements about which factors in a given cycle are pushing probability up or down, which may help couples understand why two cycles with similar headline characteristics can be assigned different estimated chances. The same logic applies to embryo ploidy prediction, where probabilities can be paired with a concise explanation of the dominant drivers such as maternal age and morphokinetics, clarifying both the value and the limits of prediction [19,21,45]. For embryo imaging, visual maps can illustrate what parts of a blastocyst image contributed most to the score and can be discussed alongside embryologist assessment. However, probabilistic outputs and visually persuasive explanations can also amplify anxiety or create false certainty if uncertainty, non-causality and the possibility of model error are not discussed explicitly [4,12].

### 7.3. Regulation, Liability and Quality Assurance

AI systems influencing embryo selection or treatment strategy should be treated as high-risk medical technologies. Current governance frameworks in Europe and the United States converge on requirements for clinical evaluation, traceable documentation of how outputs are generated and presented and ongoing post-deployment monitoring. ART-specific consensus statements extend this to the reproductive setting, emphasising independent validation of commercial tools and positioning AI as decision support under human oversight [17]. Quality assurance intersects directly with XAI because models can drift after retraining or changes in patient mix, laboratory practice, imaging conditions or protocols. Clinics, therefore, need processes not only to monitor performance but also to monitor the stability and fairness of explanations over time, including whether attribution patterns differ systematically across age groups, diagnoses or ethnic backgrounds in ways that are not clinically justified. When AI-supported decisions lead to harm, responsibility remains with the clinician, but vendors and institutions share duties around validation, maintenance, documentation and transparency [12,17].

### 7.4. Infrastructure and Sustainability

Implementation also has infrastructure and resource implications. High-resolution embryo images, time lapse video and self-supervised or generative pipelines increase demands for compute, storage and maintenance, especially when models are retrained or run continuously across centres. This raises practical questions around hosting, version control, cybersecurity and long-term support, and it introduces an environmental footprint that is increasingly relevant for healthcare systems. For ART, these constraints strengthen the case for models that are efficient, well validated and explainable in a way that supports audit and governance. In many settings, a smaller model with stable calibration and clear explanations may be preferable to a large opaque architecture that is difficult to host, update and monitor responsibly [12,17].

## 8. Knowledge Gaps and Future Directions for XAI in ART

The main limitations of the current XAI-in-ART literature can be grouped into recurring domains, each with corresponding priorities for future research and implementation. Most XAI studies in ART remain retrospective, single centre, and evaluated primarily with internal validation, often within ethnically narrow populations. Strengthening external and prospective validation across different clinics, laboratory protocols and health systems is a central next step and should include assessment not only of performance but also of whether explanations remain stable and clinically plausible across settings. Recent work has also highlighted the importance of explicitly assessing model stability and reliability in AI-based embryo selection, reinforcing the need to evaluate robustness alongside discrimination and calibration [51].

Endpoints are another consistent limitation. Implantation, biochemical pregnancy and early clinical pregnancy dominate, whereas live birth is less frequently a primary outcome, and long-term offspring outcomes are essentially absent. Future work should prioritise outcomes that matter most to patients, including cumulative live births per stimulation and, where feasible, neonatal outcomes and longer-term child health, rather than relying mainly on early surrogate endpoints [16,18].

Male factor applications remain comparatively under-developed within the XAI literature. Current examples are largely proof-of-concept studies based on small lifestyle datasets or optimisation-driven demonstrations. A clear need is explainable modelling in real andrology cohorts, including semen parameters and sperm DNA fragmentation, prediction of sperm retrieval in non-obstructive azoospermia and outcomes after varicocele intervention, anchored to clinically meaningful endpoints such as natural conception, IUI and IVF or ICSI success [24,31]. For the endometrium and receptivity, only limited work combines advanced imaging with XAI, and most studies are single centre. A priority is prospective modelling that integrates ultrasound markers, including Doppler- or contrast-enhanced perfusion indices, with molecular or histological measures, to clarify how endometrial biology interacts with stimulation dynamics and embryo characteristics [20,36]. In non-invasive ploidy and embryo viability, many studies remain modality specific. Future models should integrate morphology, time lapse kinetics and, where available, molecular signals and PGT A-related information, while providing explanations that make explicit the contribution of each level to euploidy and implantation predictions [4,45,46,47].

Most current XAI in ART remains associational. Method development should increasingly distinguish predictors from plausible intervention targets, particularly for variables that are clinically actionable such as metabolic markers, supplements and stimulation parameters. Alongside this, evaluation should move beyond discrimination to include calibration, net benefit and decision-relevant thresholds, so that clinicians can judge how an XAI-informed tool would change decisions compared with standard care [16,18]. Consistent reporting also remains a gap. ART-specific guidance for XAI studies, aligned with broader AI in medicine recommendations and ART consensus statements, would improve reproducibility by standardising reporting of data provenance, model development, explanation methods and how explanations were evaluated [12,17].

Most XAI systems remain research prototypes. Translation into practice requires integration into laboratory information systems and clinical records, with routine logging of predictions and explanations and interfaces that can be used safely in embryo selection meetings and counselling. Deployment also requires structured post-implementation monitoring to detect drift in performance and in explanation patterns across age groups, diagnoses and demographic subgroups, with predefined triggers for review, retraining or rollback [12,17]. Because no single centre is likely to cover all relevant subgroups with sufficient size, collaborative multicentre networks will be essential. Privacy-preserving approaches, including federated learning, offer a practical route to multicentre training and validation of explainable models without centralising raw embryo images or patient-level data [16,18].

## 9. Conclusions

Explainable artificial intelligence in assisted reproductive technologies is moving from an interesting add-on to a practical requirement. Across the ART workflow, from male-factor assessment and ovarian stimulation to endometrial evaluation, embryo selection and ploidy-related decision support, most studies rely on two recurring approaches. The first is feature-attribution methods for tabular models, typically used to show which clinical or laboratory variables drive predictions. The second is saliency-based visualisation for embryo or ultrasound images, used to indicate which regions appear to influence a model’s output. These tools can improve transparency and support clinical scrutiny, but they do not, by themselves, solve the main weaknesses of the current evidence base.

Most published work remains retrospective and single centre, with heterogeneous endpoints and limited reporting of calibration, clinical utility and external generalisability. Several studies demonstrate that it is feasible to attach SHAP, LIME or CAM-type explanations to cycle-level predictors and embryo imaging systems, and newer approaches attempt to move beyond heatmaps through intrinsically interpretable designs or more structured representations of embryo morphology. Even so, robust prospective evaluation and multi-centre validation are still uncommon. In this setting, explanation outputs should be read as descriptions of model behaviour within the analysed datasets, not as causal claims or instructions for intervention.

Explainability matters in ART for reasons that go beyond technical transparency. Embryo ranking and cycle-level counselling occur in emotionally and ethically sensitive contexts, where probabilistic outputs can influence expectations, decisions and trust. When used well, XAI can help clinicians explain why a prediction differs between apparently similar cycles, can highlight potential failure modes and can make bias or data limitations more visible. When used poorly, explanations can create false certainty, particularly if uncertainty and validation limits are not discussed explicitly or if visually persuasive plots are interpreted as proof of biological truth.

Progress in XAI for ART will depend less on adding new algorithms and more on improving the foundations. Three priorities stand out: first, higher-quality data with external and prospective validation across centres, laboratory protocols and patient populations, with assessment of whether explanation patterns remain stable and clinically plausible; second, evaluation that goes beyond discrimination to include calibration, decision-relevant thresholds and measures of clinical usefulness; and third, responsible implementation, including integration into real workflows, training of end users and continuous monitoring of both performance and explanation drift over time. Because no single centre is likely to represent all relevant subgroups at adequate scale, collaborative and privacy-preserving multi-centre infrastructures may be essential.

Ultimately, explainability should not be treated as a cosmetic layer added to black-box prediction, nor as a substitute for rigorous study design. Its value lies in supporting accountability, auditability and meaningful clinician–patient communication. If paired with robust validation, transparent reporting and appropriate governance, XAI has the potential to make AI-assisted reproductive care more trustworthy and clinically useful. If not, explanations risk giving undue credibility to models that remain methodologically fragile.

## Figures and Tables

**Table 1:** Tabular and clinically oriented XAI models across key decision points in ART.

Study	Clinical Task	Data Modality	Endpoint	XAI/Interpretability Approach	Validation Type	Reported Performance	Key Limitation
GhoshRoy et al. [24]	Male-factor pre-treatment screening (proof-of-concept)	Tabular (lifestyle /environment)	Semen quality category	SHAP, LIME, ELI5	Internal (cross-validation)	Accuracy > 90%; AUC near 1.0	Small dataset; internal only
Ramdass et al. [31]	Male-factor screening (proof-of-concept)	Tabular (lifestyle /environment)	Semen quality category	Global importance + similarity-based contextualisation	Internal split	Accuracy 99%	Small dataset; internal split
Wang Q. et al. [32]	Male-factor predictors linked to embryo development	Tabular (andrology /clinical)	High-quality embryo target	Interpretable modelling + causal/“what-if” intervention layer	Retrospective internal	Reported qualitatively	Retrospective single-centre
Fanton et al. [28]	Trigger timing decision support	Tabular (stimulation-day variables)	Trigger recommendation/expected MII yield	Intrinsic rule-based/linear-model interpretability	Internal test set	Comparable to more complex ML baselines; same-day MAE 2.87, R^2^ 0.64	Internal validation only
Zhang et al. [34]	Mature oocyte yield prediction at scale	Tabular (routine cycle variables)	MII oocyte count	SHAP	Temporal/independent clinical validation	RMSE 3.675; MAE 2.702; R^2^ 0.714	No external/prospective validation
Hanassab et al. [35]	Follicle-cohort optimisation on trigger day	Follicle size distribution ± basic clinical data	MII oocytes; downstream embryo yield; live birth (secondary association)	Permutation importance, TreeSHAP	Multi-centre internal-external style validation across clinics	MAE ~ 3.60; R^2^ 0.35	No fully independent external validation
Huang et al. [19]	Cycle-level outcome prediction with counselling interface	Tabular (pre-treatment + in-cycle variables)	Pregnancy outcome (as reported)	SHAP	Internal	Reported qualitatively	Internal validation only
Ejder et al. [23]	Transfer-stage prediction with modifiable exposures	Tabular (cycle + supplement exposure)	Clinical pregnancy	LIME	Internal	Reported qualitatively	Proof-of-concept/observational design
Zhu et al. [21]	PCOS-specific cycle prediction	Tabular (metabolic/androgen + cycle variables)	Live birth	SHAP	Internal validation set	AUC 0.822	Internal validation only
Wang C. et al. [36]	Endometrial perfusion-based prognostic modelling	US/CEUS-derived variables + cycle factors	Ongoing pregnancy	SHAP	Prospective single-centre	Accuracy ~ 93%; AUC ~ 0.98	Small single-centre cohort
Wan et al. [20]	Endometriosis cohort prediction	Tabular (routine clinical/cycle variables)	Clinical pregnancy	SHAP	Internal test set	AUC ~ 0.62	Moderate discrimination
Cao et al. [48]	LLM-assisted IVF outcome modelling	Tabular (IVF clinical dataset)	Clinical pregnancy	SHAP + generated explanatory text	Internal	Accuracy 0.79; AUROC 0.89	Single dataset; post hoc pipeline

Abbreviations: ART, assisted reproductive technologies; CEUS, contrast-enhanced ultrasound; IVF, in vitro fertilisation; LIME, local interpretable model-agnostic explanations; LLM, large language model; MII, metaphase II; PCOS, polycystic ovary syndrome; SHAP, Shapley additive explanations; US, ultrasound.

**Table 2:** Imaging and embryo-level XAI models for embryo assessment, selection and ploidy-related decision support. This table summarises XAI and interpretable modelling approaches applied to embryo images, time-lapse morphokinetics, embryo scoring and ploidy or post-PGT-A outcome prediction, highlighting the explainability technique and validation setting reported.

Study	Clinical Task	Data Modality	Endpoint	XAI/Interpretability Approach	Validation Type	Reported Performance	Key Limitation
Enatsu et al. [25]	Day-5 blastocyst viability prediction	Static blastocyst images ± clinical data	Clinical pregnancy; live birth	Grad-CAM (image level); SHAP (ensemble model)	Internal test set/internal CV for ensemble	AUC 0.68 image-only; AUC 0.71 ensemble; AUC 0.78 for live birth image-only	Internal validation only
Salih et al. [4]	Embryo viability prediction with multimodal fusion	Blastocyst images + clinical data	Clinical pregnancy; live birth	ScoreCAM; LRP; Bayesian reliability layer	Internal hold-out	Fusion AUC 0.91 (pregnancy)/0.88 (live birth)	Internal hold-out only
Duval et al. [26]	Time-lapse embryo ranking with clinical context	Time-lapse embryo videos + clinical data	Implantation/pregnancy-related ranking outcome	SHAP at hybrid model layer	Multi-clinic internal evaluation	Moderate discrimination with site variability	No explicit image-level XAI; variable performance across sites
Liu et al. [42]	Quantitative differentiation of similarly graded blastocysts	Static blastocyst images	Continuous ICM/TE scoring linked to live birth	Interpretable continuous ICM/TE scoring aligned with Gardner grading	Internal + external application study	AUC 0.997 (expansion), 0.903 (ICM), 0.943 (TE); significant live-birth correlation externally	Retrospective design
Kodali et al. [43]	Interpretable embryo quality classification	Static blastocyst images	Good vs. poor embryo classification	LIME superpixel explanations	Internal	Accuracy 97.7% after augmentation; LIME fidelity 0.91	Very small public dataset
Wang S. et al. [44]	Prospective AI-assisted embryo selection	Static blastocyst images with interpretable ranking support	Implantation rate; live birth and neonatal outcomes (secondary)	Interpretable AI ranking system	Prospective single-centre cohort	Implantation 80.87% vs. 68.15%	Single-centre non-randomised cohort
Luong et al. [45]	Euploidy prediction in PGT-A setting	Morphology + morphokinetics + clinical data	Euploid vs. aneuploid	SHAP; LIME	Internal + external validation cohort	Internal AUC 0.808; external AUC 0.750	Single-centre derivation with one external cohort
Ortiz et al. [46]	Post-PGT-A biochemical pregnancy loss prediction	Clinical + embryo variables	Biochemical pregnancy loss after euploid transfer	Random Forest + SHAP	Internal	Reported qualitatively	Internal validation only
Rotem et al. [47]	Latent-space visual explanation of embryo morphology	Static blastocyst images	High vs. low morphology classification	Disentangled latent axes + counterfactual editing + SHAP	Internal	Base classifier AUC 0.93; latent-feature classifier AUC 0.92	Morphology endpoint, not implantation/live birth
Yang et al. [27]	Generative/structured embryo representation	Blastocyst images	Embryo morphology representation/scoring	Latent feature attribution/structured interpretable descriptors	Internal	Reported qualitatively	Internal validation only
Wang S. et al. [37]	Automatic blastocyst evaluation with multifocal imaging	Multifocal blastocyst images	Good vs. poor blastocyst classification	Grad-CAM/CAM	Internal split	Accuracy 0.899; AUC 0.936	Morphology endpoint only
Kim H.-M. et al. [38]	Clinical pregnancy prediction with ICM/TE-enhanced images	Static blastocyst images + age	Clinical pregnancy	Grad-CAM with compartment-guided preprocessing	Internal test set	AUROC 0.741 for best enhanced model	No external validation; manual segmentation
Amitai et al. [39]	Early miscarriage prediction	Embryo morphokinetics + features	First-trimester miscarriage	Limited/no explicit XAI	Internal	Reported qualitatively	Limited explicit XAI
Arsalan et al. [40]	Blastocyst component detection	Static blastocyst microscopy images	TE/ZP/ICM/BL segmentation	Grad-CAM visualisation	Internal test set	Mean Jaccard index 87.0%	Segmentation task, not clinical outcome prediction
Boucret et al. [41]	Time-lapse selection among matched high-quality embryos	Time-lapse embryo videos	Implantation in KID-based setting	Limited/no explicit XAI in model itself	Internal hold-out/internal CV	AUC 0.64 in clinically relevant task; Brier 0.30	Limited explicit XAI; moderate discrimination

Abbreviations: CAM, class activation map; Grad-CAM, gradient-weighted class activation mapping; ICM, inner cell mass; LIME, local interpretable model-agnostic explanations; LRP, layer-wise relevance propagation; PGT-A, preimplantation genetic testing for aneuploidy; SHAP, Shapley additive explanations; TE, trophectoderm.

## Data Availability

No new data were created or analyzed in this study.
